# The morphological pathogenesis of isolated superior mesenteric artery dissection

**DOI:** 10.3389/fcvm.2025.1653988

**Published:** 2025-10-10

**Authors:** Li Hou, Keli Yin, Jiang Xiong, Chengxin Weng, Jiarong Wang, Yuhan Qi, Tinghui Zheng, Tiehao Wang, Ding Yuan

**Affiliations:** ^1^ Day Surgery Centre, General Practice Medical Centre, West China Hospital, Sichuan University, Chengdu, China; ^2^ Department of Mechanics & Engineering, College Architecture & Environment, Sichuan University, Chengdu, China; ^3^ Department of Vascular and Endovascular Surgery, The First Medical Centre, Chinese PLA General Hospital, Beijing, China; ^4^ Division of Vascular Surgery, Department of General Surgery, West China Hospital, Sichuan University, Chengdu, China; ^5^ Department of Applied Mechanics, Sichuan University, Chengdu, China

**Keywords:** superior mesenteric artery, isolated dissection, anatomy, pathogenesis, etiology

## Abstract

**Background:**

Isolated superior mesenteric artery dissection (ISMAD) is a rare arterial disease, and its exact cause is still not well understood. This study aimed to investigate the potential role of anatomical factors in the development of ISMAD.

**Methods:**

This case-control study included patients diagnosed with ISMAD via computed tomography angiography from two major medical centers in China. An equal number of age-sex and body mass index matched patients without aortic and superior mesenteric artery disease were selected as controls. Several anatomical parameters were compared between the ISMAD group and the control group. Significant parameters were identified through univariate and multivariate analyses, and models were evaluated using receiver operating characteristic (ROC) curve analysis. A *p*-value < 0.05 was considered statistically significant.

**Results:**

A total of 60 patients with isolated superior mesenteric artery dissection and 60 age-sex (52.6 ± 6.1 vs. 52.2 ± 13.5, *p* = 0.82) and body mass index (24.3 ± 2.5 vs. 24.0 ± 4.0, *p* = 0.72) matched normal controls from two major hospitals in China were included in the study. Compared with normal controls, the multivariate analysis revealed that curvature (OR 1.239, 95% CI 1.122–1.369, p < 0.001) and tortuosity (OR 0.002, 95% CI, 0.000–0.083, *p* = 0.001) were independent predictors of ISMAD occurrence.

**Conclusion:**

Patients with ISMAD exhibited higher levels of curvature and lower levels of tortuosity compared to normal control group.

## Introduction

Isolated superior mesenteric artery dissection (ISMAD) refers to a vascular lesion formed by spontaneous rupture of the intima of the superior mesenteric artery (SMA), causing blood flow to enter between the intima and media of the blood vessel wall through the intimal rupture without involvement of the aorta, which may potentially result in impaired blood flow, ischemia, or rupture. ISMAD is a rare condition that was once considered an uncommon entity but has been increasingly recognized with the widespread use of computed tomography angiography (CTA).

As the detection rate of ISMAD has improved, researchers have increasingly focused on the disease, leading to the proposal of relevant classifications (1–7). Understanding the factors that contribute to the development of ISMAD is crucial for improving diagnosis and management strategies. While atherosclerosis and connective tissue diseases are commonly associated with aortic dissection, their significance in the etiology of ISMAD is less pronounced (8, 9). ISMAD is more common in young and middle-aged individuals without significant underlying diseases. The condition of the vessel wall (such as vascular diseases and connective tissue disorders) is not the primary influencing factor. In contrast, hemodynamic factors play a more crucial role (10). Previous studies have suggested that various factors, including hemodynamics, anatomical parameters, and genetic predispositions, may play a role in the development of ISMAD. However, these studies have not comprehensively addressed the interaction between anatomical anomalies and hemodynamic alterations (11–13).

While the mentioned studies have shed light on certain factors related to the formation of ISMAD, it is important to note that the morphological and anatomical factors specific to the mesentery itself have not been extensively supported by the available data. Previous morphological parameters have been based on the simple two-dimensional structure of the SMA, in fact, the SMA trunk morphology is similar to the aorta and has a specific three-dimensional morphology, which has not been paid attention to in previous studies. This study aims to investigate the potential role of anatomical and hemodynamic factors in the development of ISMAD. Specifically, we seek to answer the following research questions: 1) What anatomical parameters are significantly associated with the occurrence of ISMAD? 2) How do hemodynamic alterations, resulting from these anatomical parameters, contribute to the pathogenesis of ISMAD? By addressing these questions, we proposed two concepts: curvature (which reflects the curvature of the SMA path) and tortuosity (which reflects the spatial tortuosity of the SMA path). We hope to provide a more comprehensive understanding of the factors that predispose individuals to ISMAD and to highlight the importance of considering both anatomical and hemodynamic factors in its pathogenesis.

## Materials and methods

The Agency's Review Board approved the two-center study (2021-1152), which was conducted in accordance with the ethical standards of the Agency's Committee on Human Experimentation and reported under the Enhanced Observational Epidemiological Research Report (STROBE) guidelines. The work has been reported in line with the STROCSS criteria (14).

### Study population

**Figure 1:**
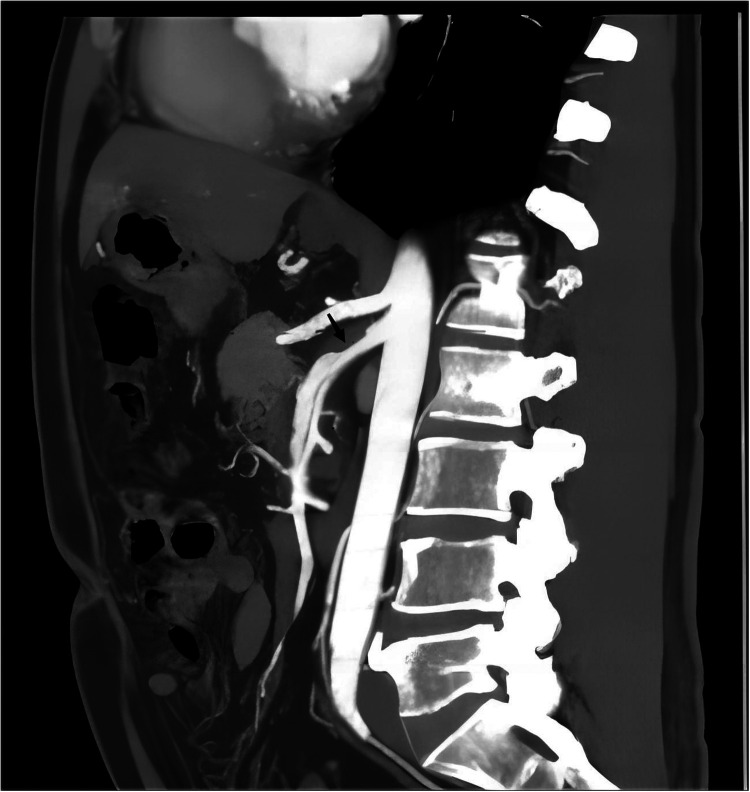
The typical performance of ISMAD in the sagittal plane of a computed tomography scan, with the black arrow pointing to the location of the dissection.

This study is a two-center case control study. The study included a total of 60 patients diagnosed with ISMAD using CTA (Figure 1). Diagnosis of ISMAD was confirmed when one of the following signs was seen in the SMA on the initial CT scan: (1) intimal flap and contrast enhancement within the false lumen; (2) crescent-shaped area along the wall of the SMA with higher attenuation than blood, showing no contrast enhancement after intravenous administration of contrast material (7, 15, 16). In our screening of patients with superior mesenteric artery dissection, we excluded those with a history of aortic dissection, major abdominal trauma, endovascular procedures for SMA, superior mesenteric artery embolism or thrombosis, connective tissue disorders, vasculitis, or intramural hematoma of the superior mesenteric artery. Finally, 60 patients constituted the study population. Clinical information of each patient was obtained including demographics, symptoms, comorbidities, treatment, and outcomes from the electric medical records. Among them, 41 patients were recruited from our center, and an additional 19 patients were recruited from another center. The control group for this study consisted of 60 individuals from our center with normal superior mesenteric artery and aorta. The age, sex and BMI of the control group were matched with ISMAD group. Their anatomical data, obtained through CTA, were included for comparative analysis.

### Measurement of anatomic data

**Figure 2:**
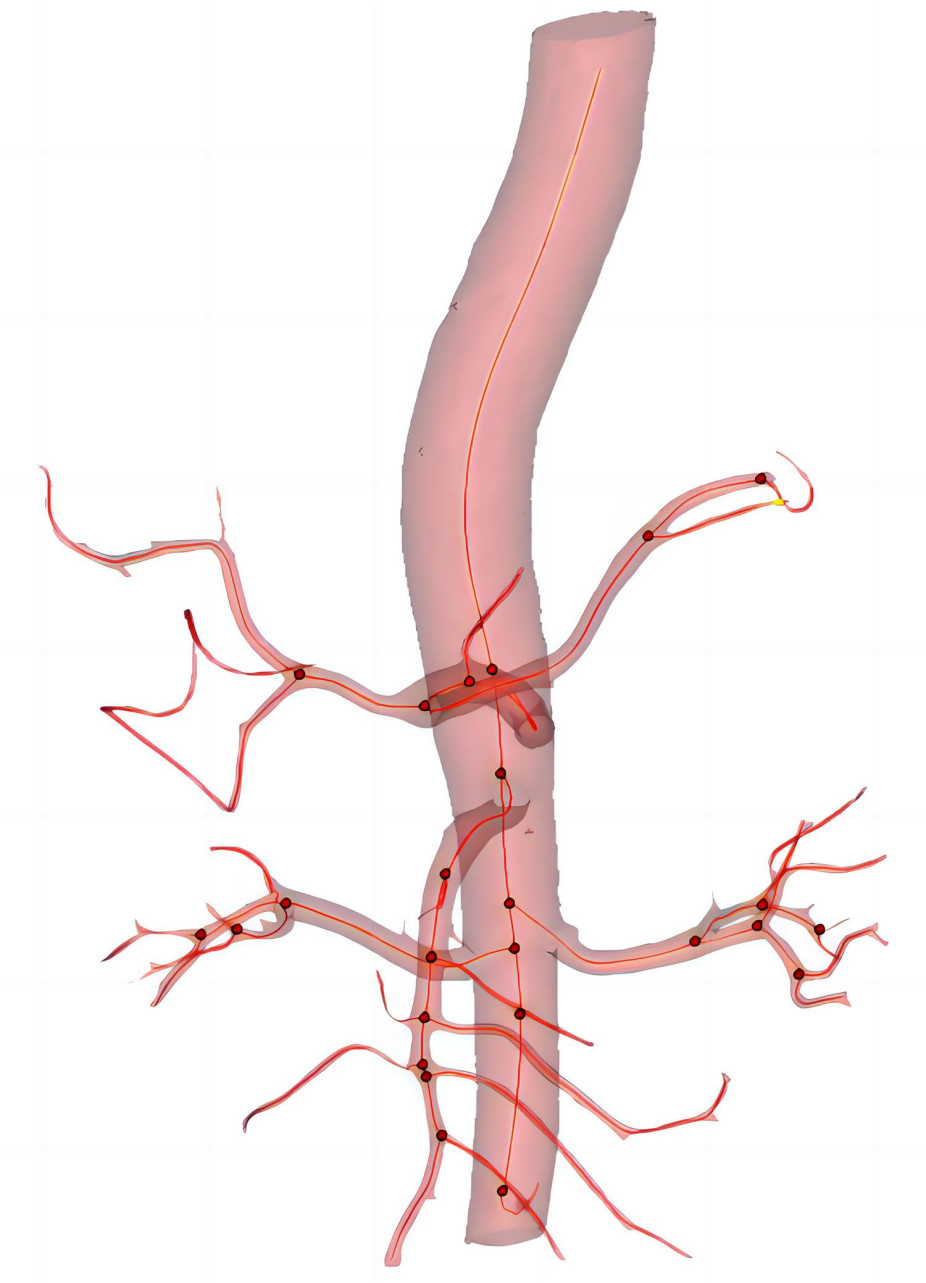
The three-dimension reconstruction of image and center line (red line), red dot indicates the presence of branched artery.

In this study, all anatomical parameter measurements were performed using Materialise's interactive medical image control system software (Materialise, Plymouth, Mich). The specific method is as follows: the patient's CT images are imported into the MIMICS software system for the reconstruction of the aorta and its main branch arteries. Using the diameter measurement tool provided by the software, measurements are taken while delineating the centerline of the arterial lumen. The curvature and curvature are measured along this centerline (Figure 2). All reconstructions and measurements were performed individually by two researchers.

The main anatomical parameters assessed in this study included: 1) angle between the SMA and the aorta (*α* Angle); 2) diameter of the entrance of SMA; 3) diameter of the aorta 1 centimeter above the celiac trunk artery (CA); 4) diameter of the aorta at CA plane; 5) curvature of the SMA and 6) tortuosity of the SMA.

**Figure 3:**
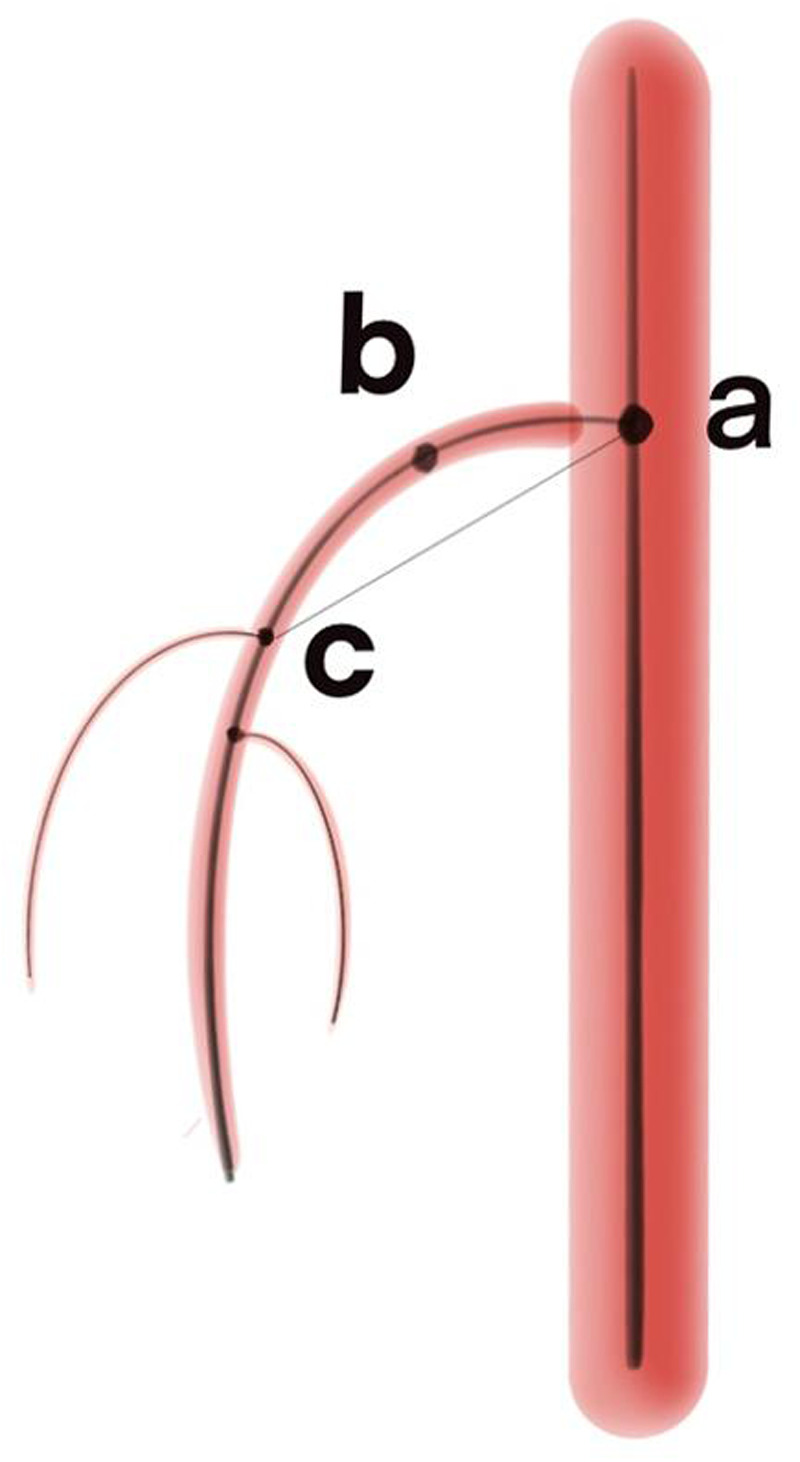
The schematic diagram of the definitions of curvature and tortuosity. Point a is the center line intersection point between superior mesenteric artery and the aorta; Point b represents the maximum curvature point on the centerline; Point c is the center line intersection point between superior mesenteric artery and superior mesenteric artery’s first branch.

Below are some definitions of concepts used in this article:

1. *α* angle: The Angle formed between the SMA and the aorta: the intersection point between the center line of the left renal artery and the center line of the aorta was the first point, the intersection point between the center line of the SMA and the center line of the aorta was the second point, and the maximum curvature point of the center line of SMA was the third point. The spatial coordinates of the three points were taken, and the Angle was calculated by vector operation.
2. Diameter of the entrance of SMA: The “best fit diameter measuring” tool was used in the Mimics to measure the diameter of the entrance of the SMA over the center line.
3. Diameter of the aorta 1 centimeter above the celiac artery (CA): The “best fit diameter measuring” tool was used in the Mimics to measure the diameter of the aorta 1 centimeter above the CA over the center line.
4. Diameter of the aorta at CA plane: The “best fit diameter measuring” tool was used in the Mimics to measure the diameter of the aorta at CA plane.
5. Curvature: By establishing the centerline of the SMA, select the maximum curvature from the centerline to define curvature (point b in the Figure 3). Since the effect size is too large, we chose to multiply it by 100 times to present it. Curvature reflects the curvature of SMA.
6. Tortuosity: The center lines intersection point between SMA and the aorta is the first point (point *a* in the Figure 3), and the center line intersection point between SMA and SMA's first branch is the second point (point *c* in the Figure 3). The ratio of the curve distance to the straight-line distance between these two points. This concept reflects the degree of twisting of the SMA.

### Statistical analysis

In this study, statistical analysis was performed using IBM SPSS Statistics for Windows, version 26.0 (IBM Corp., ARMONK, N.Y., USA) and GraphPad Prisma version 9.0 (GraphPad Software, San Diego, California USA). Bland–Altman analysis was performed to evaluate the inter- and intra-observer variability. Student's *t* test, the Wilcoxon rank-sum test, and Chi-square test were used to compare the results of the study population. Subsequently, a combined model was developed by curvature and tortuosity using binary logistic regression analysis. The area under a receiver operating characteristic (ROC) curve (AUC) was utilized to compare the predictive effect of the curvature model, tortuosity, and the combined model. Effect sizes were indicated by odds ratios (OR) with corresponding 95% confidence intervals (CI). A significance level of *p* < 0.05 was considered statistically significant.

## Results

This study enrolled 60 patients with ISMAD and 60 individuals in the normal control group. These two groups of patients are comparable in terms of age (52.6 ± 6.1 vs. 52.2 ± 13.5, *p* = 0.82), gender (56 vs. 51, *p* = 0.24), body mass index (24.3 ± 2.5 vs. 24.0 ± 4.0, *p* = 0.70) and hypertension (9 vs. 11, *p* = 0,624). Other comorbidities such as smoking (21 vs. 17, *p* = 0.432) and diabetes (0 vs. 3, *p* = 0.244) were not statistically different between the two groups (Table 1).

**Table 1:** Baseline characteristics and results of univariate analysis in of ISMAD and control group.

Features	ISMAD (*n* = 60)	Controls (*n* = 60)	*P* value
Baseline characteristics			
Male, *n* (%)	56 (93%)	51 (85%)	0.240
Age, mean ± SD, years	52.6 ± 6.1	52.2 ± 13.5	0.820
BMI, mean ± SD (kg/m^2^)	24.3 ± 2.5	24.0 ± 4.0	0.702
Hypertension, *n* (%)	9 (15%)	11 (18%)	0.624
Diabetes, *n* (%)	0 (0%)	3 (5%)	0.244
Smoker, *n* (%)	21 (35%)	17 (28%)	0.432
Anatomical data			
*α* angle, mean ± SD,°	77.4 ± 14.8	68.5 ± 17.5	0.003
Diameter of the entrance of the SMA, mean ± SD, (mm)	8.5 ± 1.3	7.6 ± 1.5	0.001
Diameter of aorta at SMA plane, mean ± SD, (mm)	19.8 ± 1.9	19.0 ± 2.7	0.054
Diameter of the aorta 1 cm above CA, mean ± SD, (mm)	20.9 ± 2.1	19.6 ± 3.0	0.008
Diameter of the aorta at CA plane, mean ± SD, (mm)	21.0 ± 2.0	19.8 ± 2.8	0.007
Curvature, median (IQR)	16 (13.75)	9 (2)	0.001
Tortuosity, median (IQR)	1.19 (0.18)	1.27 (0.21)	0.011

ISMAD, isolated superior mesenteric artery dissection; SMA: superior mesenteric artery, CA: celiac trunk artery.

The study findings revealed significant differences in anatomical parameters between patients with ISMAD and the normal control group. Patients with ISMAD exhibited larger values in the following parameters compared to the normal control group: *α* angle (77.4 ± 14.8 vs. 68.5 ± 17.5, *p* = 0.003), diameter of the entrance of the SMA (8.5 ± 1.3 mm vs. 7.6 ± 1.5 mm, *p* = 0.001), diameter of the aorta 1 centimeter above CA (20.9 ± 2.1 mm vs. 19.6 ± 3.0 mm, *p* = 0.008), diameter of the aorta at CA plane (21.0 ± 2.0 mm vs. 19.8 ± 2.8 mm, *p* < 0.05), curvature (16 vs. 9, *p* = 0.001). ISMAD patients had lower tortuosity of the SMA (1.19 vs. 1.27, *p* = 0.011) compared to the normal control group. However, no statistically significant differences were observed in the aorta at SMA plane (19.8 ± 1.9 mm vs. 19.0 ± 2.7 mm, *p* = 0.054) (Table 1). The Bland–Altman analysis demonstrated no significant differences between the measurements obtained by the two researchers (Supplementary Materials).

After conducting univariate analysis, several anatomical parameters were selected for multivariate logistic regression analysis, including *α* angle, diameter of the entrance of the SMA, diameter of the aorta 1 centimeter above CA, diameter of the aorta at CA plane, curvature and tortuosity. The multivariate analysis revealed that curvature (radian) (OR: 1.239, 95% CI: 1.122–1.369, *p* < 0.05) and tortuosity (spatial walking tortuosity) (OR: 0.002, 95% CI: 0.000–0.083, *p* < 0.05) were independent predictors of ISMAD occurrence. *α* angle, diameter of the entrance of the SMA, diameter of the aorta 1 centimeter above CA, diameter of the aorta at CA plane did not show independent association with ISMAD (Table 2).

**Table 2:** Multivariate logistic regression analysis of anatomic parameters in ISMAD patients and normal controls.

Anatomical parameters	OR	95%CI	*P* value
α angle	1.022	0.98–1.07	0.322
Diameter of the entrance of the SMA	0.963	0.636–1.458	0.859
Diameter of the aorta 1 cm above CA	0.972	0.393–2.409	0.952
Diameter of the aorta at CA plane	1.396	0.526–3.700	0.503
Curvature	1.239	1.122–1.369	<0.001
Tortuosity	0.002	0.000–0.083	0.001

ISMAD, isolated superior mesenteric artery dissection; SMA, superior mesenteric artery; CA, celiac trunk artery; CI, confidence interval.

**Figure 4:**
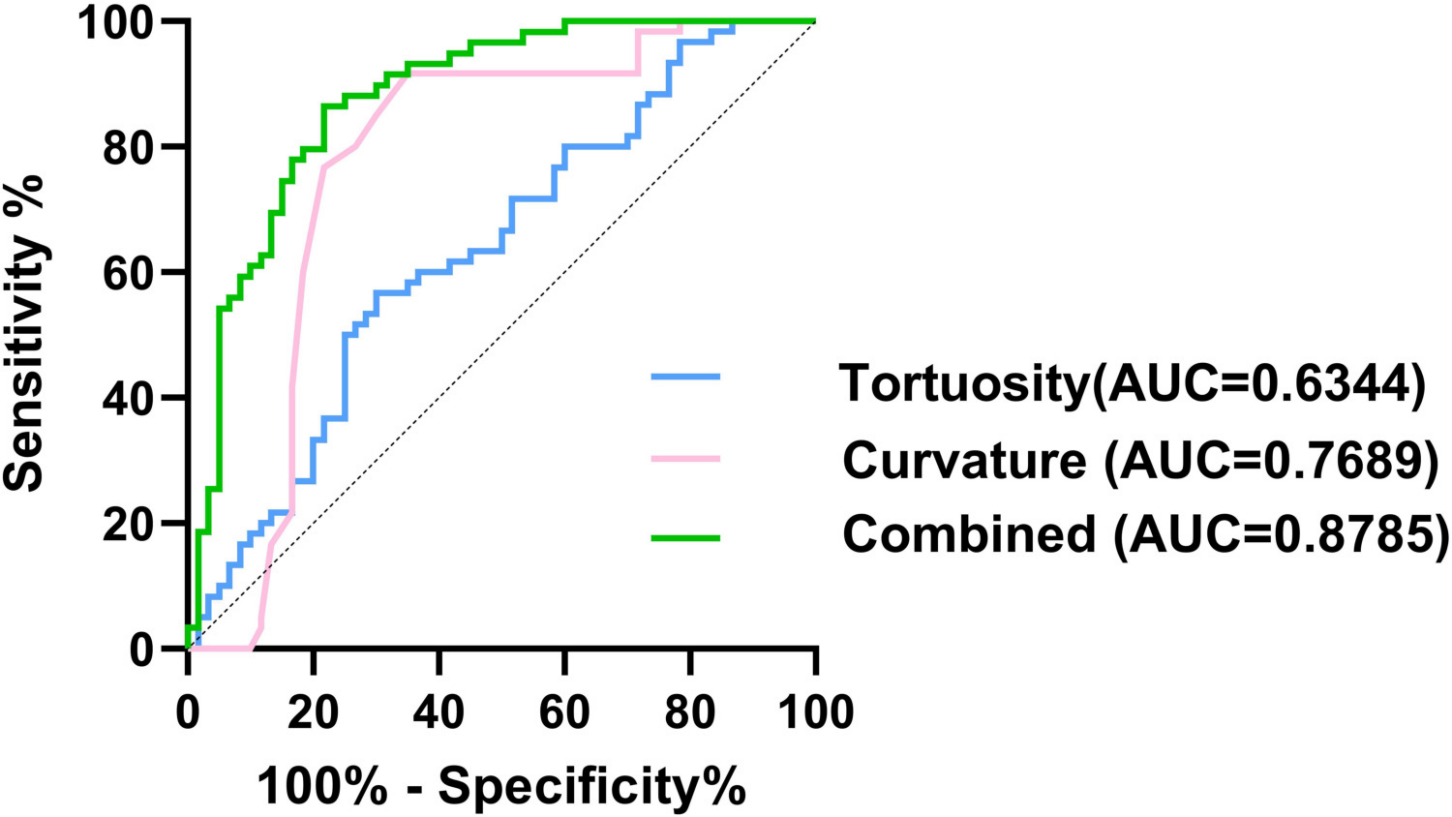
Comparison of ROC curves of the tortuosity (blue line), curvature (pink line), and combined model (green line).

To assess their predictive value, ROC curves were constructed, showing that the area under the curve (AUC) for curvature was 0.769 (95% CI: 0.675–0.863) with a corresponding specificity of 0.65 and sensitivity of 0.92, and the AUC for tortuosity was 0.634 (95% CI: 0.535–0.734) with a specificity of 0.7 and sensitivity of 0.57 (Figure 4). Further analysis involved the selection of independent associated factors through multifactor analysis, with curvature and tortuosity used to construct an ISMAD independent associated factor model. The ROC analysis demonstrated that the AUC of this model was 0.879 (95% CI: 0.816–0.941), with a specificity of 0.86 and sensitivity of 0.78, indicating a significantly better association with ISMAD compared to the two independent factors.

## Discussion

The global epidemiology of ISMAD exhibits significant geographical disparities, with a notably higher observed frequency in Asian populations compared to Caucasian groups. Most published clinical studies on ISMAD originate from Asian countries such as China, South Korea, and Japan, suggesting a higher disease burden in these regions. This observation is supported by comparative prevalence data: a South Korean study conducted among symptomatic patients presenting to the emergency department reported an IMAD prevalence of 0.96%, which starkly contrasts with the 0.03% prevalence found in a French study (17–19). Regarding the treatment of ISMAD, asymptomatic patients do not require specific treatment and only need blood pressure control. Conservative treatment is effective for the vast majority of symptomatic patients (17). The issue of whether to administer anticoagulation has always been controversial, and a systematic review of observational studies indicates that anticoagulation therapy provides no greater benefit to patients with superior mesenteric artery dissection who are undergoing conservative treatment (20). Antiplatelet therapy is recommended for patients with ISMAD (19). Vigilant clinical monitoring is imperative for SMAD patients, with particular attention to signs of mesenteric ischemia. When bowel ischemia is suspected, endovascular intervention emerges as the primary therapeutic approach if the anatomical characteristics allowed. The latest meta-analysis demonstrates an 88% success rate of lumen remodeling in endovascular treatment for ISMAD (21). When endovascular intervention is unfeasible or unsuccessful, prompt open surgery remains the definitive therapeutic solution.

The exact etiology and pathogenesis of ISMAD remain unclear. Studies have indicated that approximately 90% of ISMAD patients are male (22), suggesting gender as a potential risk factor for the disease. A meta-analysis including 80 studies with a total of 1040 patients revealed that among patients with ISMAD, the prevalence of smoking and hypertension was 43% and 41%, respectively (23)*.* Differences in hemodynamics and anatomical structures have also been proposed as potential causes of the dissection (10, 11). In a previous study (11), anatomical parameters of 38 dissection patients were measured and compared with normal patients. They proposed that an increased angle between the SMA and the abdominal aorta may be an anatomical factor contributing to the occurrence of dissection. Because the greater the angle between the inner wall of the convex surface of the SMA and the distal aorta, the higher the shear stress. Another study by Park et al. (10), identified hemodynamic abnormalities at the transition point from the fixed segment of SMA to the relative moving segment as the main cause of SMA detachment. They simulated SMA hemodynamics and analyzed the relationship between the angle between SMA and the abdominal aorta and blood flow. They suggested that ISMAD development is likely associated with the convex curvature of SMA caused by abnormal hemodynamic forces. Abnormal mechanical stress was observed in the anatomical anterior wall of SMA in their computational simulation model.

Like previous study, the angle between the aorta and the SMA was also measured in this study. In previous studies, the angle between the aorta and SMA was measured directly from the sagittal plane of CTA, some from the central lumen, and some along the vascular wall. The determination of the two lines to determine the angle was artificial and subjective and may not reflect the real blood flow in the vessel. Instead, we chose to reconstruct the center line in the lumen and find three points from the center line that best reflect the angle between SMA and aorta. It was found that the *α* angle in ISMAD patients was larger than that in the normal control group, and the difference was statistically significant in univariate analysis, consistent with Wu et al.'s findings. However, in multivariate analysis, this parameter did not show statistical significance. This finding suggests that we may have exaggerated the role of this angle in the formation of dissection in previous studies, leading to misleading conclusions. it is important to recognize that SMA is a three-dimensional structure and considering only the angle between the SMA and the aorta may not provide a comprehensive understanding of the anatomical factors involved in ISMAD. The angle represents a simplified two-dimensional perspective and may not fully capture the complexity and variability of the SMA's anatomy. Therefore, we need to reassess the actual role of this angle in dissection formation and conduct a comprehensive analysis by incorporating other three-dimensional factors and hemodynamics to obtain more accurate conclusions.

In this study, the curvature we defined is similar to the convexity described by Park et al., both describing the curvature of the SMA. We measured the maximum curvature of the SMA from its central line and compared it with the normal control group. We found that the curvature in ISMAD patients was larger than in the normal control group, indicating a more severe bending of the SMA. This finding further supports Park's hypothesis that the occurrence of ISMAD is related to the convexity and curvature of SMA based on real data.

Additionally, this study introduced the new conception of tortuosity, which represent the twisting degree during SMA movement. The results showed that the degree of tortuosity in ISMAD patients was smaller compared to normal patients. We speculate that this may be related to the effect of swirling flow, which refers to blood flow within the blood vessels that exhibits a single-vortex swirling pattern. Spiral blood flow is not only present in the aorta but also in other locations such as the right coronary artery, descending aorta, iliac arteries, and femoral arteries (24–26). It enhances the transport of oxygen from the blood to the arterial wall and reduces the adhesion of blood cells to the arterial intima (27–29). These effects of spiral blood flow can alleviate the burden on arteries and protect them from the pathological effects of atherosclerosis, thrombus formation, and intimal hyperplasia. In terms of hemodynamics, the presence of swirling flow can balance the uneven distribution of shear stress and contribute to a more stable blood flow (28, 30). In ISMAD patients, the lack of rotational flow may disrupt blood flow and result in uneven distribution of wall shear stress, predisposing to dissection.

Overall, this study provides insights into the anatomical and hemodynamic factors associated with ISMAD. The findings highlight the importance of considering factors such as curvature and tortuosity in understanding the pathogenesis of ISMAD and its potential relationship with hemodynamic abnormalities. Our speculation is that regions with greater curvature in the SMA may experience higher pressure, resulting in uneven pressure distribution and increased wall shear force. These factors may contribute to the development of ISMAD. Ongoing studies in our center aim to further investigate the hemodynamic involved in the formation of this dissection. By examining the hemodynamic aspects, we hope to gain a deeper understanding of the mechanisms underlying ISMAD and contribute to the development of effective diagnostic and therapeutic approaches.

It is important to recognize that this study primarily focuses on exploring the potential anatomical factors contributing to the development of superior mesenteric artery dissection (ISMAD). The hemodynamic changes induced by anatomical factors, as well as other concomitant conditions (such as elevated blood pressure), require further investigation in subsequent studies. The study has several limitations that should be acknowledged. Firstly, the sample size was relatively small, which may limit the generalizability of the findings. Larger-scale studies are necessary to confirm these results and provide more robust evidence. Secondly, as the independent reconstruction of true and false cavities is difficult, the presence of dissection in patients with ISMAD may affect the accuracy of the reconstructed centerline. Thirdly, the study design was retrospective and observational, which may introduce selection bias and limit the ability to establish causality between the anatomical and hemodynamic factors and ISMAD, Prospective studies are needed to validate these associations and explore the temporal relationship between these factors and the development of ISMAD. Lastly, this study focused solely on anatomical parameter anomalies and did not explore the associated hemodynamic changes, which warrants further investigation.

## Conclusion

Due to the limitations of imaging technology in the past, the incidence of isolated superior mesenteric artery as a vascular dissection lesion is underestimated. ISMAD is more common in young and middle-aged individuals without significant underlying diseases. its pathogenesis needs to be further explored, and this study found that patients with ISMAD exhibited higher levels of curvature and lower levels of tortuosity. The importance of *α* angle in the pathogenesis of ISMAD is limited. These findings suggest that alterations in hemodynamics resulting from these anatomical factors may potentially contribute to the development of mesenteric dissection.

## Data Availability

The raw data supporting the conclusions of this article will be made available by the authors, without undue reservation.

## Supplementary material

Supplementary Figure 1 Bland–Altman analysis results for curvature.

Supplementary Figure 2 Bland–Altman analysis results for Diameter of the aorta 1 centimeter above the celiac artery.

Supplementary Figure 3 Bland–Altman analysis results for Diameter of the aorta at CA plane.

Supplementary Figure 4 Bland–Altman analysis results for Diameter of the entrance of SMA.

Supplementary Figure 5 Bland–Altman analysis results for Diameter of Tortuosity.

Supplementary Figure 6 Bland–Altman analysis results for Diameter of α angle.
