# Supplementary figures and images for: The morphological pathogenesis of isolated superior mesenteric artery dissection
Source: Front Cardiovasc Med. 2025 Oct 10;12:1653988. doi: 10.3389/fcvm.2025.1653988 (PMC12549576; doi:10.3389/fcvm.2025.1653988)

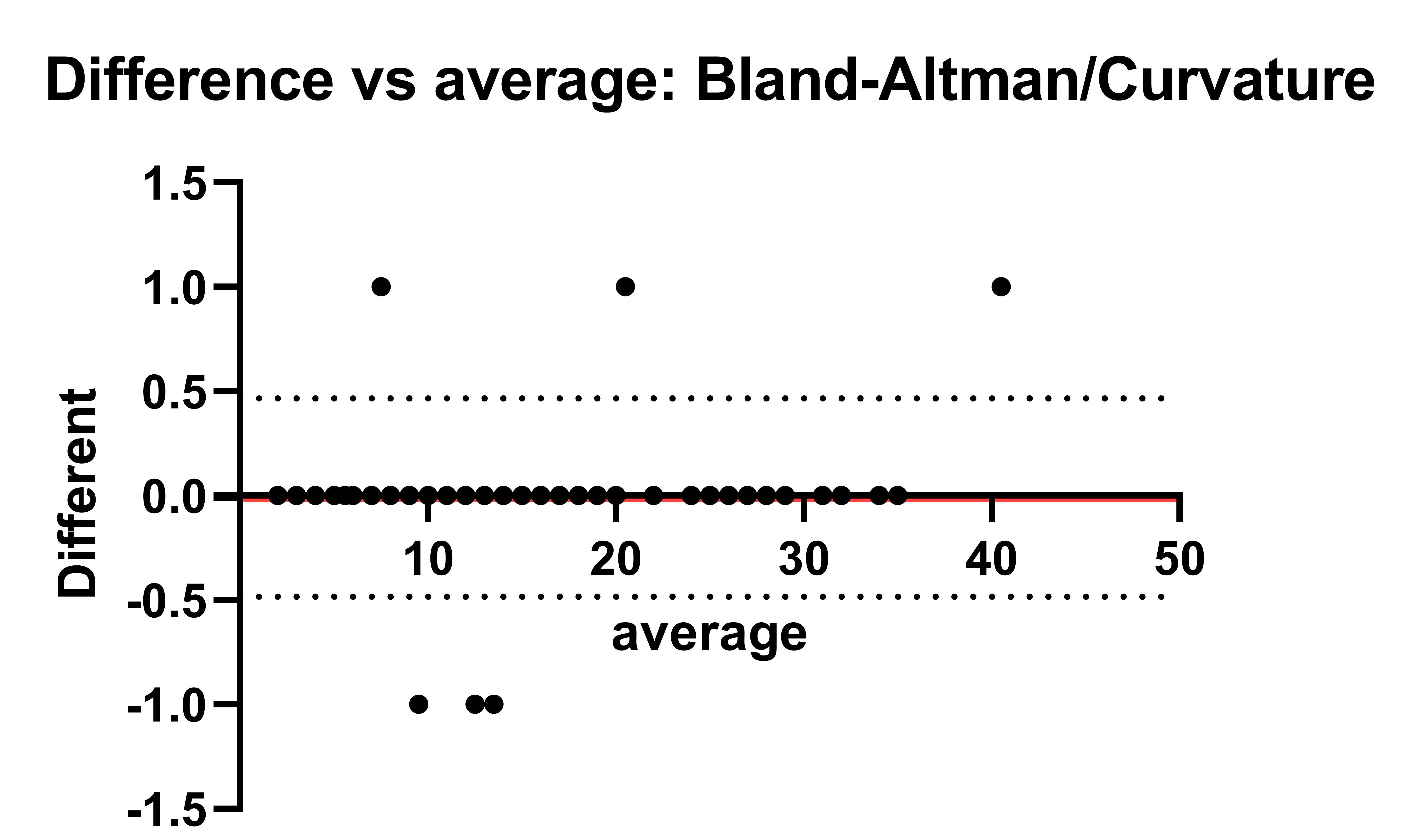

Supplement: Supplementary Figure 1 — Bland–Altman analysis results for curvature. [file Image1.jpeg]

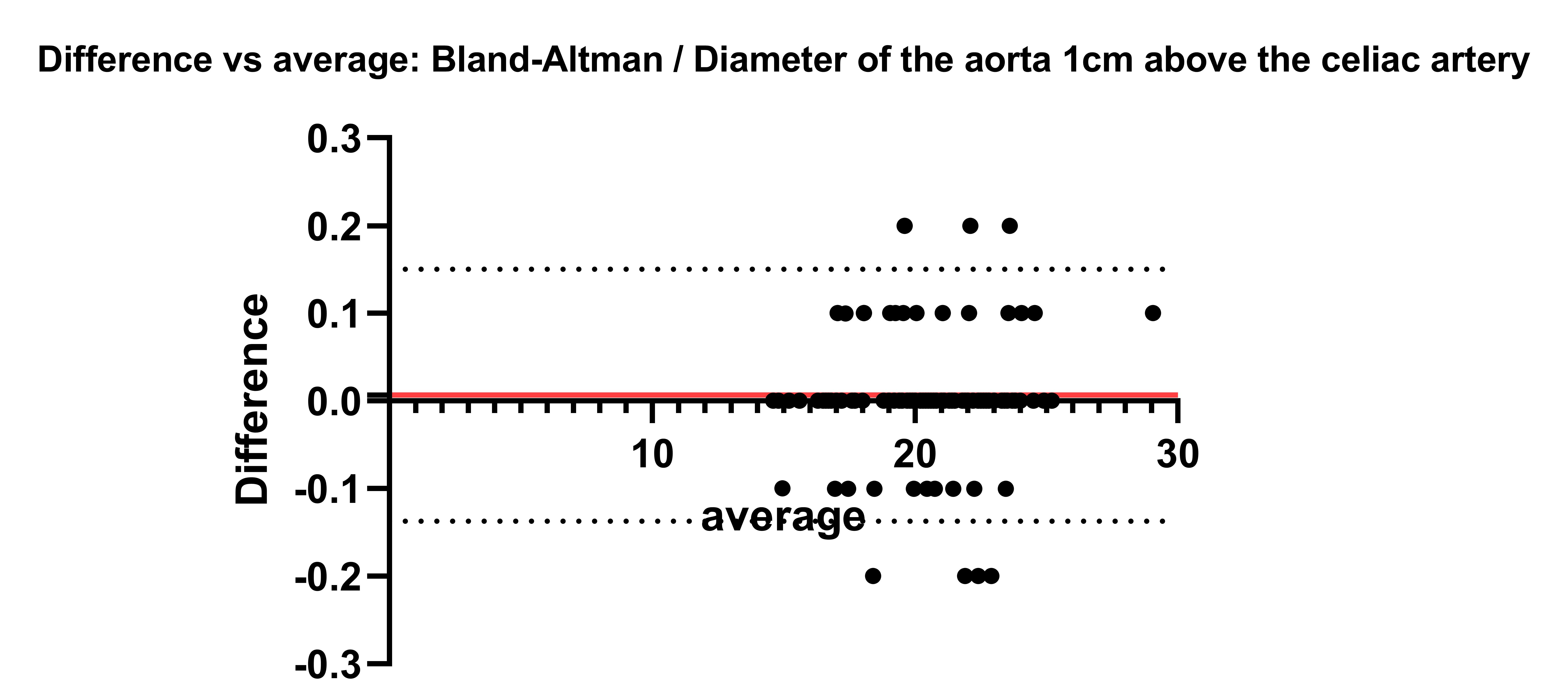

Supplement: Supplementary Figure 2 — Bland–Altman analysis results for Diameter of the aorta 1 centimeter above the celiac artery. [file Image2.jpeg]

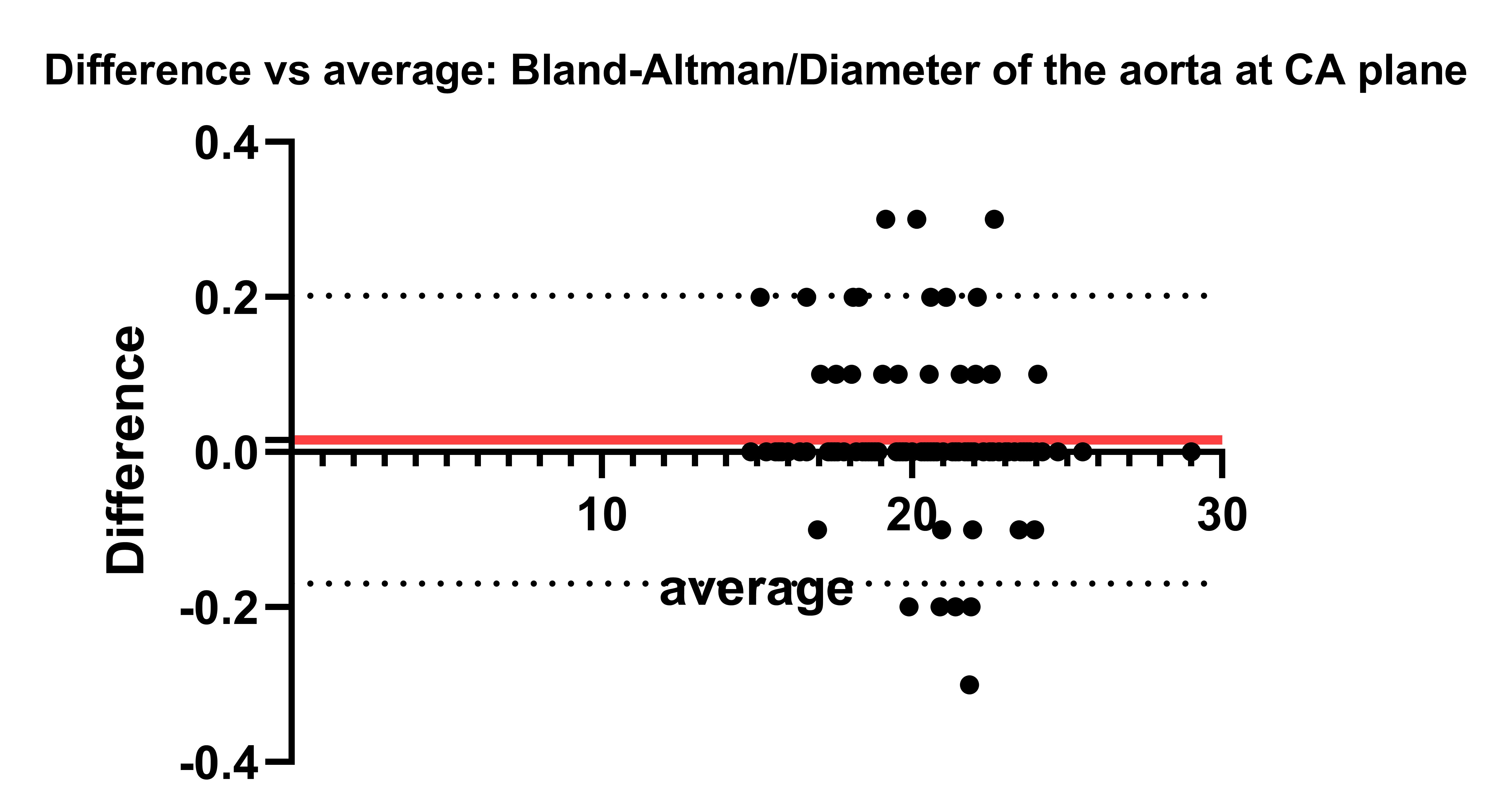

Supplement: Supplementary Figure 3 — Bland–Altman analysis results for Diameter of the aorta at CA plane. [file Image3.jpeg]

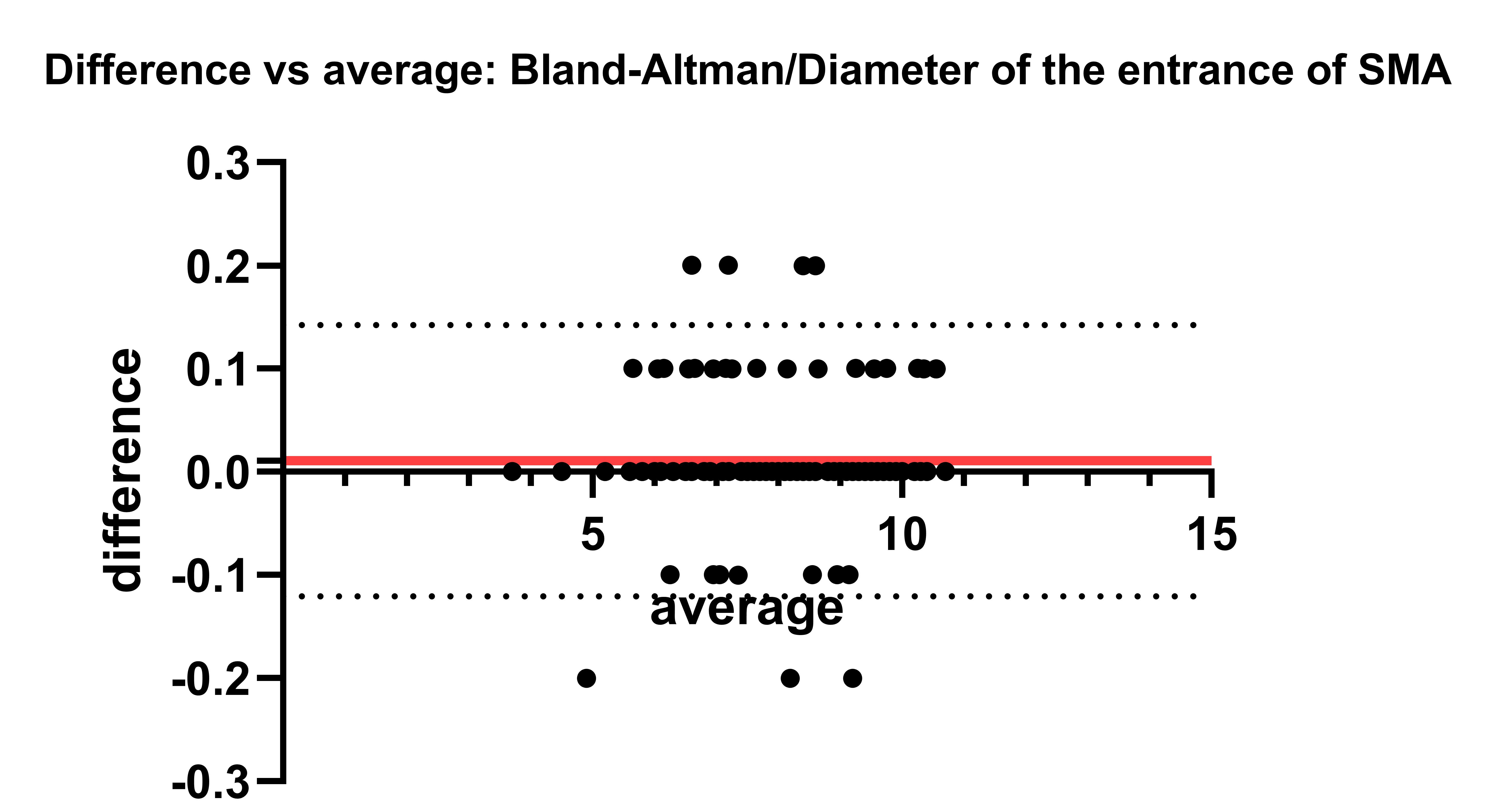

Supplement: Supplementary Figure 4 — Bland–Altman analysis results for Diameter of the entrance of SMA. [file Image4.jpeg]

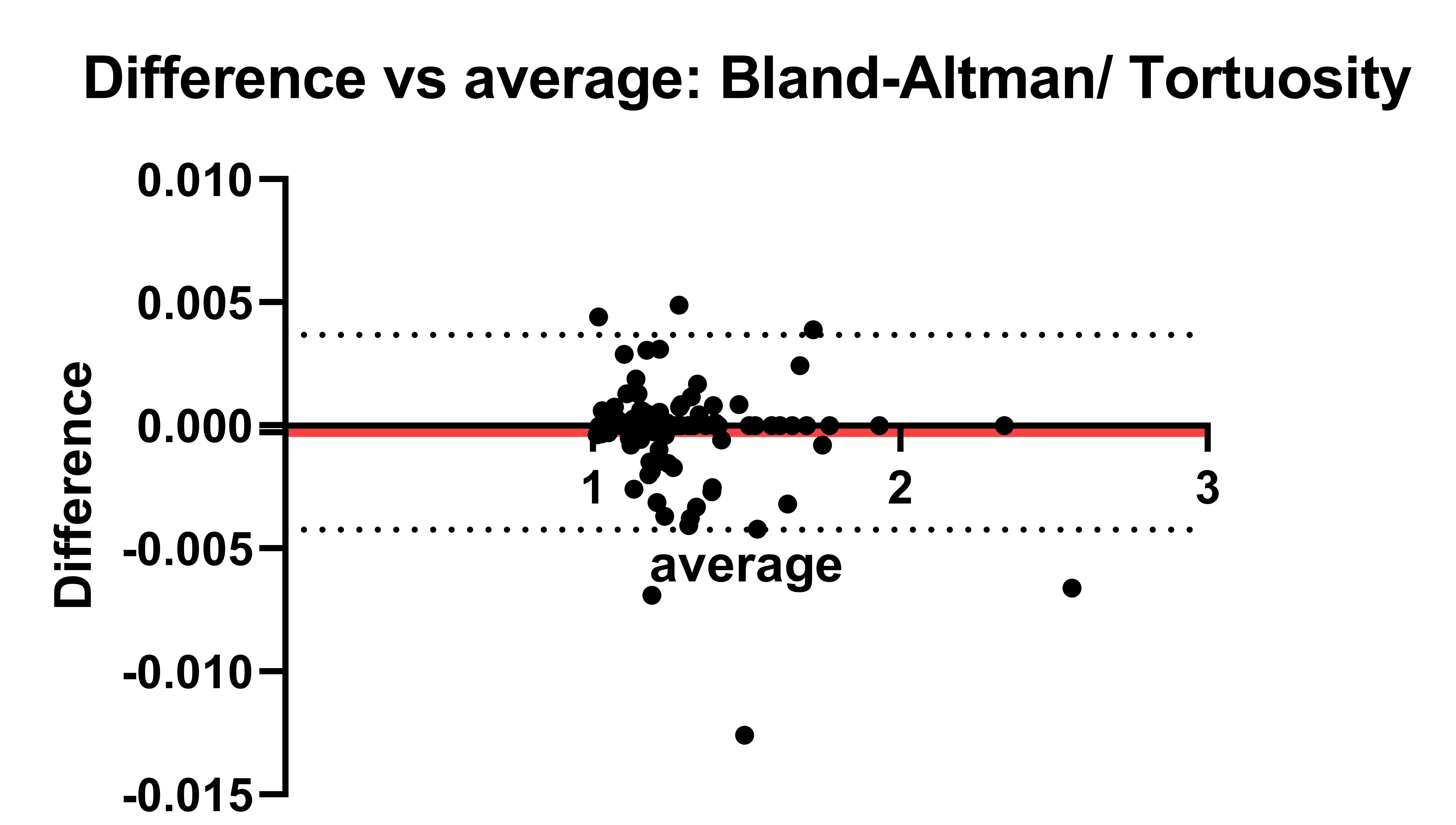

Supplement: Supplementary Figure 5 — Bland–Altman analysis results for Diameter of Tortuosity. [file Image5.jpeg]

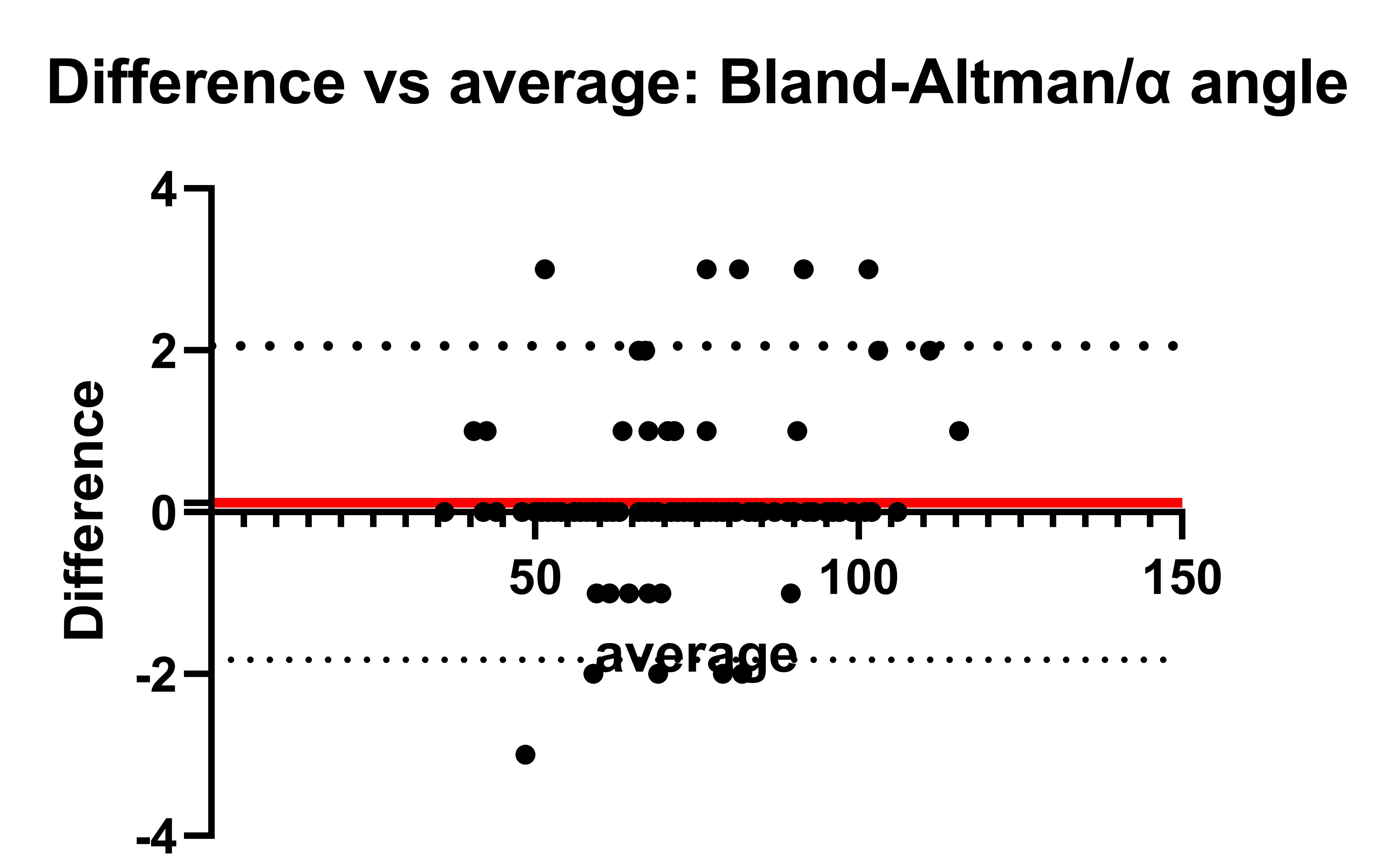

Supplement: Supplementary Figure 6 — Bland–Altman analysis results for Diameter of α angle. [file Image6.jpeg]
